# Mussel Inspired Chemistry and Bacteria Derived Polymers for Oral Mucosal Adhesion and Drug Delivery

**DOI:** 10.3389/fbioe.2021.663764

**Published:** 2021-05-05

**Authors:** Nazanin Owji, Nandin Mandakhbayar, David A. Gregory, Elena Marcello, Hae-won Kim, Ipsita Roy, Jonathan C. Knowles

**Affiliations:** ^1^Division of Biomaterials and Tissue Engineering, Royal Free Hospital, Eastman Dental Institute, University College London, London, United Kingdom; ^2^Institute of Tissue Regeneration Engineering (ITREN), Dankook University, Cheonan, South Korea; ^3^Department of Materials Science and Engineering, University of Sheffield, Sheffield, United Kingdom; ^4^Faculty of Science and Technology, University of Westminster, London, United Kingdom; ^5^Department of Nanobiomedical Science, BK21 Nanobiomedicine (NBM) Global Research Center for Regenerative Medicine, Dankook University, Cheonan, South Korea; ^6^Department of Biomaterials Science, School of Dentistry, Dankook University, Cheonan, South Korea; ^7^University College London (UCL) Eastman-Korea Dental Medicine Innovation Centre, Dankook University, Cheonan, South Korea

**Keywords:** biomimetic, surface functionalization, polydopamine chemistry, drug delivery, oral mucosa, polyhydroxyalkanoates

## Abstract

Ulceration of the oral mucosa is common, can arise at any age and as a consequence of the pain lessens enjoyment and quality of life. Current treatment options often involve the use of topical corticosteroids with poor drug delivery systems and inadequate contact time. In order to achieve local controlled delivery to the lesion with optimal adhesion, we utilized a simple polydopamine chemistry technique inspired by mussels to replicate their adhesive functionality. This was coupled with production of a group of naturally produced polymers, known as polyhydroxyalkanoates (PHA) as the delivery system. Initial work focused on the synthesis of PHA using *Pseudomonas mendocina* CH50; once synthesized and extracted from the bacteria, the PHAs were solvent processed into films. Polydopamine coating was subsequently achieved by immersing the solvent cast film in a polymerized dopamine solution. Fourier Transform Infrared Spectroscopy (FTIR) spectroscopy confirmed functionalization of the PHA films via the presence of amine groups. Further characterization of the samples was carried out via surface energy measurements and Scanning Electron Microscopy (SEM) micrographs for surface topography. An adhesion test via reverse compression testing directly assessed adhesive properties and revealed an increase in polydopamine coated samples. To further identify the effect of surface coating, LIVE/DEAD imaging and Alamar Blue metabolic activity evaluated attachment and proliferation of fibroblasts on the biofilm surfaces, with higher cell growth in favor of the coated samples. Finally, *in vivo* biocompatibility was investigated in a rat model where the polydopamine coated PHA showed less inflammatory response over time compared to uncoated samples with sign of neovascularization. In conclusion, this simple mussel inspired polydopamine chemistry introduces a step change in bio-surface functionalization and holds great promise for the treatment of oral conditions.

## Graphical Abstract

Illustration of PHA synthesis followed by a simple muscle inspired chemistry polydopamine coating.

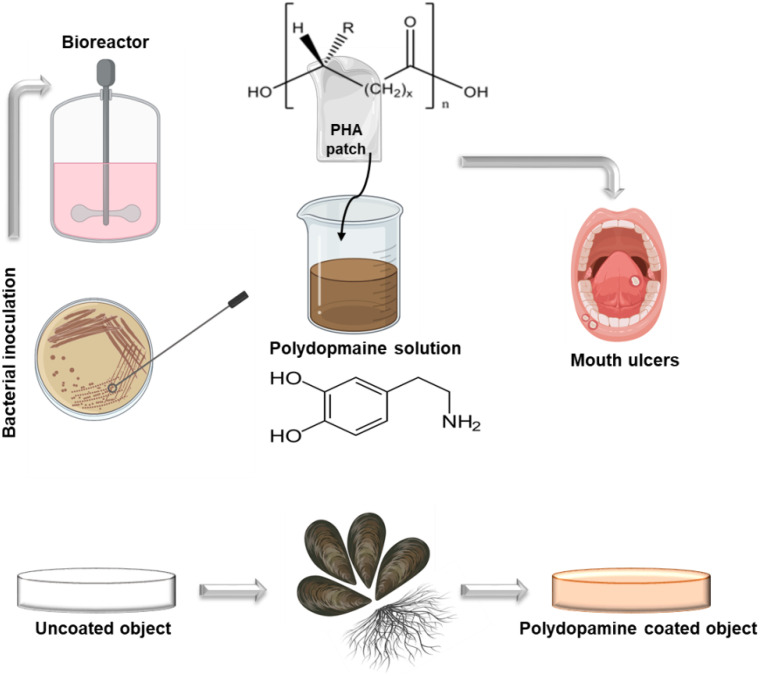

## Introduction

Oral lichen planus and recurrent aphthous ulcers are common lesions that affect the mucosal lining of the oral cavity. This causes substantial morbidity in a reported 25% of the world’s population at some point in their lifetime (Scully and Carrozzo, 2008). The pathogenesis of both conditions is not entirely understood, and they lack effective clinical management. This in turn adversely impacts the ability to speak, swallow and ultimately lessens the quality of life (Paderni et al., 2012). The mainstay of therapy of such disorders remains topical corticosteroids that distribute the treatment over the entire mouth and only offer short contact times between the drug and lesion (Gupta et al., 2017). Furthermore, higher risk of fungal infection such as candidiasis, has been reported to be associated with corticosteroids (van Boven et al., 2013). Therefore, there is a need for a delivery system that is easy to apply and wear, can be retained on the oral mucosa and most importantly allows delivery of different corticosteroids in a controlled manner. In oral medicine, however, attaching membranes to wet surfaces offers significant challenges; this is due to the continuous flow of saliva and mechanical stresses within the oral cavity (Jones et al., 2009). Additionally, this washes away the active substances leading to shorter exposure times and unpredictable drug distribution.

In recent years advances in polymer science and surface functionalization has led to an increasing interest in the fabrication of muco-adhesive patches and films for targeted drug delivery. These include synthetic polymers such as hydroxypropylmethylcellulose, polymethacrylate derivatives and polyacrylic acids (PAA) as well as naturally occurring polymers such as hyaluronic acid and chitosan. Amongst the above mentioned polymers, synthetic polyacrylic acid has attracted significant amount of attention as a bioadhesive polymeric system (Salamat-Miller et al., 2005); Shajaei and Xiaoling designed and characterized a copolymer of PAA and polyethylene glycol (PEG) monoethyl ether mono methacrylate (PAA-co-PEG) for exhibiting optimal buccal adhesion (Kumar et al., 2014). Similarly, Lele Hoffman investigated novel polymers of PAA complexed with PEGylated drug conjugates (Schattling et al., 2017). However, the high glass transition temperature (*T*_g_) of PAA suggests decreased interpenetration at initial stages of adhesion while the polymer is still in its glassy state (Kutyla et al., 2013). Moreover, this can lead to sub-optimal wetting which makes it a rather poor delivery system. There has also been a current interest in mucoadhesive electrospun fiber-based technologies (Asencio et al., 2018; Colley et al., 2018), however, to date there remains no means of achieving local controlled delivery of corticosteroids to the oral mucosa. The only adhesive type of devices developed are inert and with poor patient compliance due to low efficacy in both adherence and feel.

In this work we have utilized a novel and simple chemistry used by mussels to attach to a wide variety of wet surfaces; in nature, mussels (Bivalvia) adhere strongly in wet conditions of high shear stress from water flow, which is achieved by secreting different adhesive proteins (Numata and Baker, 2014; Ahn, 2017). It has been shown that these proteins contain a substantial amount of 3,4-dihydroxy-L-phenylalanine (DOPA) and lysine amino acids in their sequence (Numata and Baker, 2014). This has consequently led to the understanding that co-existence of catechol (DOPA) and amine (lysine) groups is key in demonstrating strong adhesion. Polydopamine (PD), containing both catechol and amine groups, was discovered as a surface coating method in 2007 and has since obtained great interest in the biomedical field for surface modification of a wide range of materials (Lee et al., 2007). Our aim was to use the dopamine self-polymerization to investigate the feasibility of this unique chemistry on a family of naturally produced, bacterially synthesized polymers, Polyhydroxyalkanoates (PHA), for drug delivery applications targeted to the oral mucosa. PHAs are a group of polyesters produced by microorganisms under nutrient limiting growth conditions (Chen and Wu, 2005). Properties such as biodegradability and thermo-processability has put PHAs in a prominent place in the vast field of biomaterial science. In recent years, alternation in PHA monomeric structure has facilitated production of biocompatible polymers with highly specific mechanical properties, for applications in both conventional medical devices to tissue engineering and drug delivery (Zhao F. et al., 2020). Based on the monomer unit, PHAs are classified into two main types: short chain length PHAs, with monomer chain length varying between C_4_-C_5_ (*scl*-PHAs); and medium chain length PHAs (*mcl*-PHAs), with monomer chain length varying between C_6_-C_16_ (Kim et al., 2020). The *mcl*-PHAs are highly elastomeric in nature (Basnett et al., 2017); this includes Poly (3-hydroxyoctanoate-*co*-3-hydroxydecanoate-*co*-3-hydroxydodecanoate), P(3HO-*co*-3HD-*co*-3HDD), which was used in this work. The use of this novel of PHA holds significant promise in the biomedical field, especially in soft tissue repair by converting a cheap carbon source, to a high−value medical product (Shahid et al., 2021). Moreover, properties such as hydrophobicity, can be enhanced by PD coating as a simple surface functionalization approach for the ultimate aim of targeted drug delivery in oral medicine.

## Materials and Methods

### Production of P(3HO-*co*-3HD-*co*-3HDD) by *Pseudomonas mendocina* CH50

Initial synthesis and production of PHAs was carried out by *Pseudomonas mendocina* CH50 in 20 g/L coconut oil as the main carbon source (Basnett et al., 2018). This was done by using a 15 L bioreactor in batch mode. The temporal profile of the production was obtained by monitoring optical density (OD_450_), biomass, nitrogen, pH and dissolved oxygen tension (%DOT) throughout the course of the fermentation. Nitrogen was measured using the phenol hypochlorite method. PHA was recovered from the freeze-dried biomass via a two-stage Soxhlet extraction method using methanol and chloroform. The polymer was then precipitated in ice-cold methanol under stirred conditions. Structural characterization of the P(3HO-*co*-3HD-*co*-3HDD) was carried out using ^13^C-NMR and ^1^H- NMR (Bruker Avance III 600 Cryo). The monomeric composition was investigated through Gas Chromatography Mass Spectrometry (GC-MS) analysis (Chrompack CP-3800 and a Saturn 2000 MS/MS) on methanolyzed samples of the produced PHAs, as described by Lukasiewicz et al. (2018).

The P(3HO-*co*-3HD-*co*-3HDD) was then solvent processed into films for the aim of surface functionalization; for this a solvent casting method was used to dissolve 5 weight percent of the polymer in chloroform, cast into glass petri dishes and left in the fume hood to dry overnight.

### Polydopamine Coating

**FIGURE 1:**
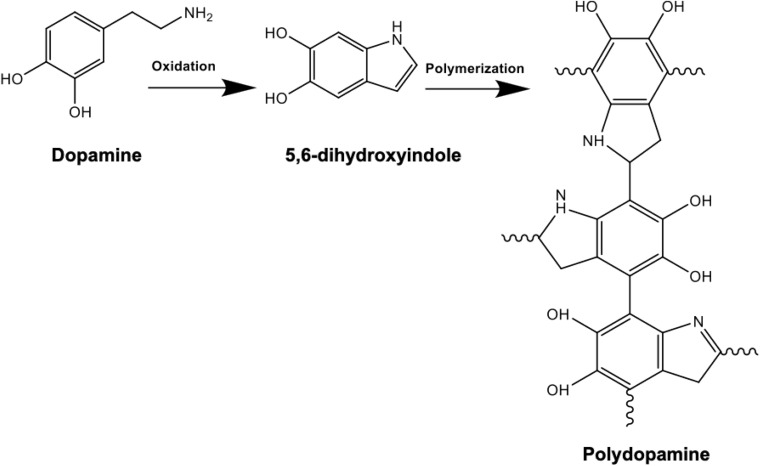
Chemical reaction schematic of polydopamine composed of indole and dopamine units: polymerization is achieved following dissolving 2 mg/ml of dopamine in 10 mM TRIS buffer at an alkaline pH of 8.5. Successful PD coating in this oxidation reaction can be confirmed by the presence of N-H as a result of surface functionalization.

Following the synthesis and purification of PHAs, the solvent casted films were dried for PD surface functionalization. Dopamine solution was prepared by dissolving 2 mg/ml of dopamine in 10 mM TRIS buffer where addition of NaOH helped reach an alkaline pH of 8.5 (Lee et al., 2009). Surface coating of the PHA films was then accomplished by immersion of the samples in the prepared alkaline PD solution. Dopamine monomers were then subjected to self-initiated polymerization where spontaneous deposition of a conforming coating occurred, as shown in Figure 1. During the 24-h incubation period, the polydopamine layer was formed on the surface. The PHA films were subsequently washed with distilled water in order to eliminate the unreacted dopamine monomers and dried at 37°C (Guo et al., 2020). Finally, the samples were stored in a desiccator filled with silica beads to avoid exposure to humidity.

### Fourier Transform Infrared Spectroscopy (FTIR)

The successful coating of PD was investigated via FTIR spectroscopy to detect the presence and absorbance values of amine peaks. Spectra were obtained by placing the PHA films in contact with the Attenuated Total Reflection (ATR). Spectrum software v 5.0.1 (Perkin Elmer, Beaconsfield, United Kingdom) identified the peak intensities of each chemical group; the wavenumber range was 500–4,000 cm^–1^ with resolution of 4 cm^–1^.

### Contact Angle Measurement

In order to assess the hydrophilic/hydrophobic nature of the samples and determine the surface free energy, contact angle measurements were recorded. This was performed using a CAM 200 Optical Contact Angle Meter instrument (KSV, Finland) where a needle was filled with three different liquids including water, glycerol and diiodomethane and located above the center of the sample. To establish the surface-liquid interface, the measurement was carried out after placement of a drop of liquid on the PHA surfaces, before and after PD coating, with a circular algorithm technique. Subsequently, the angle was investigated in the CAM 200 analysis software. In this technique, Young’s Dupre equation was used to evaluate the sum of polar and dispersive components to generate automated surface free energy values.

### Adhesion Testing via Reverse Compression Mechanical Analysis

The adhesive property of the PHA films, before and after coating, was evaluated using a dynamic mechanical analyzer (DMA; Discovery DMA850, TA Instruments, New Castle, United States) at room temperature. The samples were cut 11 mm in diameter with a thickness value of 0.29 mm. Reverse compression testing was subsequently conducted with a pre-load value of 1 N and a ramp rate of 0.1 mm/min. Following initial contact between the PHA films and the compression plate, a displacement value of −25 mm was inserted which resulted in detachment of the plate from the samples. To determine the adhesive properties of the samples, stress and strain values were calculated accordingly.

### Cell Culture

Human fibroblasts [Institute of Regenerative Medicine at the Texas A&M Health Science Centre College of Medicine (United States)] were cultured in polystyrene flasks in minimum essential medium (α-MEM, Gibco BRL) supplemented with 10% fetal bovine serum (FBS) (Invitrogen) and 100 U/ml of penicillin/streptomycin (P/S) (Sigma-Aldrich). They were subsequently incubated at 37°C, 5% CO_2_ and harvested for experiments once they reached 80% confluence at passage 4.

### Metabolic Activity

PHA films were placed in-24 well tissue culture plates (Thermo Fisher Scientific, Loughborough, United Kingdom). A total of 10,000 cells per disc were seeded in each well and incubated at 37°C 5% CO_2_. To determine cell proliferation, at day 1, 4, and 7 of culture, 100 μl of Alamar Blue dye (Alamar Blue, ABD Serotrec) was added to each well and incubated for a 4-h period. Fluorescence values (excitation wavelength of 530 nm and emission wavelength of 590 nm) were then measured using a Fluroscan Ascent plate reader (Labsystems, Helsinki, Finland).

### LIVE and DEAD Imaging

Following the seeding of fibroblasts on PHA films, they were placed in a 24 well tissue culture plate in α-MEM and incubated at 37°C, 5% CO_2_ for 1, 4 and 7 days. At each time point, the discs were washed with phosphate buffer solution (PBS) and a mixture of 2.5 μl Calcein + 5 μl EthD-1 (LIVE/DEAD^®^ Viability/Cytotoxicity Kit for mammalian cells, Thermo Fisher Scientific Inc.) was added to the well. The samples were then incubated in a petri dish, immersed in PBS for 20 min in the dark to remove any excess fluorophore and visualized under a confocal laser scanning microscope (Leica DM IRE2, Leica, Germany).

### Scanning Electron Microscopy (SEM) Images

Samples were fixed in 3% glutaraldehyde and 0.1 M cacodylate buffer and stored at 4°C overnight. Serial ethyl alcohol dehydration was carried out the next day for 10 min followed by immersing the polymer films in hexamethyldisilazane and leaving them in the hood for 1 h to dry. Finally, PHA films were coated with 95% gold and 5% palladium (Polaron E5000 Sputter Coater, Quoram Technologies, Laughton, United Kingdom) and SEM (Philips XL30 Field Emission SEM, Amsterdam, Netherlands) was used to visualize the surface of the specimen discs.

### *In vivo* Subcutaneous Implantation

**FIGURE 2:**
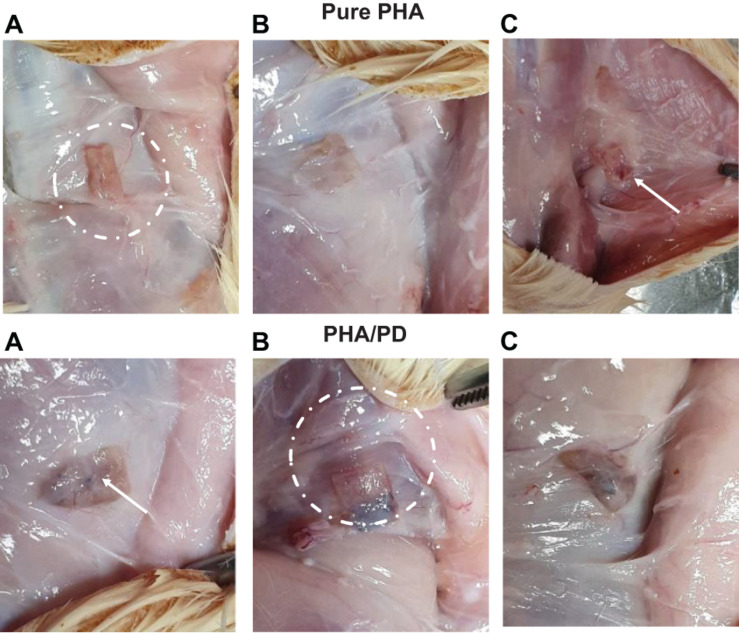
The above figure illustrates time-lapse images the implantation of PHA and PHA/PD patches in a rat’s model. The polymer film was initially inserted subcutaneously where the positioning can be clearly observed in **(A,B)**. The final suturing is also shown prior to retrieval of the specimens after 2 and 4 weeks where the state of the tissues was stable with no visible signs of inflammation or fibrous invasions **(C)**.

The effect of PD coating was evaluated *in vivo* in 10-week-old male Sprague–Dawley rats; the rats were shaved and prepared for implant insertion by performing a thorough diiodine wash, the PHA films (*n* = 3) were then inserted subcutaneously at a suitable depth to avoid any unnecessary movement. Post implantation of the films, suturing of the incision was completed and the rats were transferred to a controlled environment with fixed temperature and humidity. Finally, for the aim of histological examination, the rats were sacrificed 2- and 4-week post implantation (Figure 2).

### Hematoxylin and Eosin (H&E)

The harvested tissue sections were embedded in paraffin wax for histological sectioning and analysis; the serial paraffin sections, cut to 5 μm thickness, were deparaffinized and stained by haematoxylin and eosin (H&E). A light microscope (Olympus BX50) was used to capture images of the sections. For further analysis and processing, Image-Pro Plus (Media Cybernetics, United States) and ImageJ were used, respectively.

### CD31 Immunostaining

For immunohistochemical staining, the deparaffinized sections were washed with PBS and incubated overnight at 4°C with CD31 (Santacruz, United States) antibody. After rinsing with PBS, the sections were incubated in AlexaFluor488-conjugated secondary antibody (Cell Signaling, United States) in a humidified chamber for 1 h at room temperature. The nucleus was stained with DAPI. The fluorescence images were observed and analyzed by confocal laser scanning microscope, Zeiss LSM 510 (Carl Zeiss, Germany).

### Statistical Analysis

The results were statistically analyzed using one-way analysis of variance (ANOVA) with Tukey’s *post hoc* test; *p* < 0.05 was considered to be statistically significant.

## Results

**FIGURE 3:**
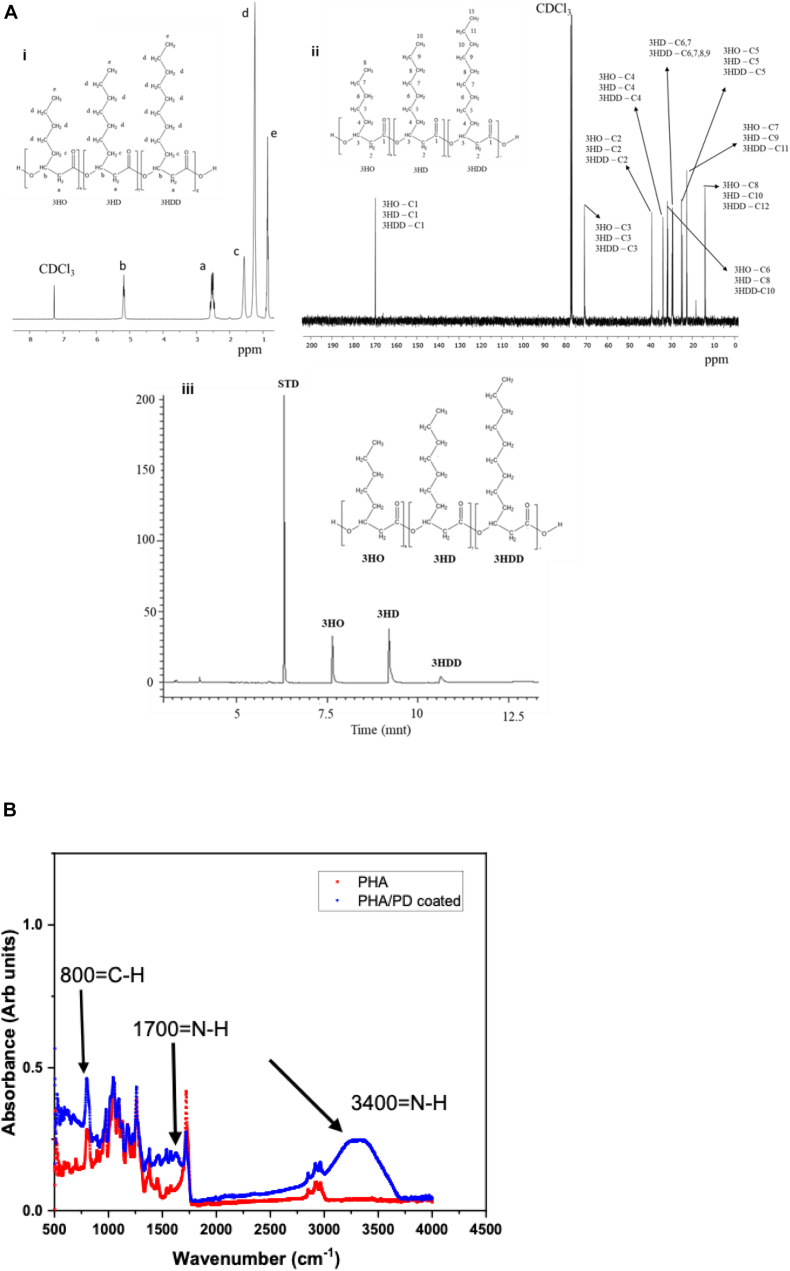
**(Ai)** representative ^1^H-NMR and ^13^C-NMR spectra of P(3HO-*co*-3HD-*co*-3HDD). The chemical structure of both polymers is shown in each figure, indicating the assignment of each peak to the corresponding proton (a) and carbon (b) atom in the molecule. **(Aiii)** shows the GC-MS chromatogram of P(3HO-*co*-3HD-*co*-3HDD); the peaks at retention time 7.6, 9.2, and 10.6 min were identified as the methyl esters of 3-hydroxyoctanoic acid (3HO), 3-hydroxydecanoic acid (3HD), and 3-hydroxydodecanoic acid (3HDD), respectively. Methyl benzoate was used as the internal standard (STD). Finally, **(B)** presents Fourier Transform Infrared Spectroscopy spectra of the PHA and PHA/PD coated films; the broad peak at 3,400 cm^–1^ and 1700 cm^–1^ identifies the presence of a strong N-H stretch, which consequently confirms successful surface functionalization with the PD coating.

The chemical structure of P(3HO-*co*-3HD-*co*-3HDD) was confirmed through ^1^H and ^13^C NMR, as shown in Figure 3. The ^1^H-NMR spectra showed five peaks typical of medium chain length PHAs while the ^13^C NMR indicated the presence of 30 peaks (Figures 3Ai,ii). GC-MS analysis was then used to determine the monomeric composition of the polymer: three peaks were detected at 6.7, 9.2, and 10.6 min, as shown in Figure 3Aiii. These peaks were identified as the methyl ester of 3-hydroxyoctanoic acid, 3-hydroxydecanoic acid and 3-hydroxydodecanoic acid, respectively. Therefore, the polymer produced was identified as P(3HO-*co*-3HD-*co*-3HDD), in agreement with the data reported by Basnett et al. (2018).

Following the chemical analysis of the synthesized P(3HO-*co*-3HD-*co*-3HDD) using *Pseudomonas mendocina* CH50, solvent cast PHA films were fabricated and immersed in a PD solution. In order to determine the efficacy of the surface coating, FTIR spectroscopy was used to investigate initial functionalization via production of amine groups (Yang et al., 2015). The broad peak at 3,400 correlates with an N-H amine stretch (Foo et al., 2017) and can instantly confirm successful coating with PD, shown in Figure 3. Similarly, an amide group is present at 1,700 cm^–1^ post modification as well as higher absorbance of C-H groups at 800 cm^–1^compared to pure PHA.

**FIGURE 4:**
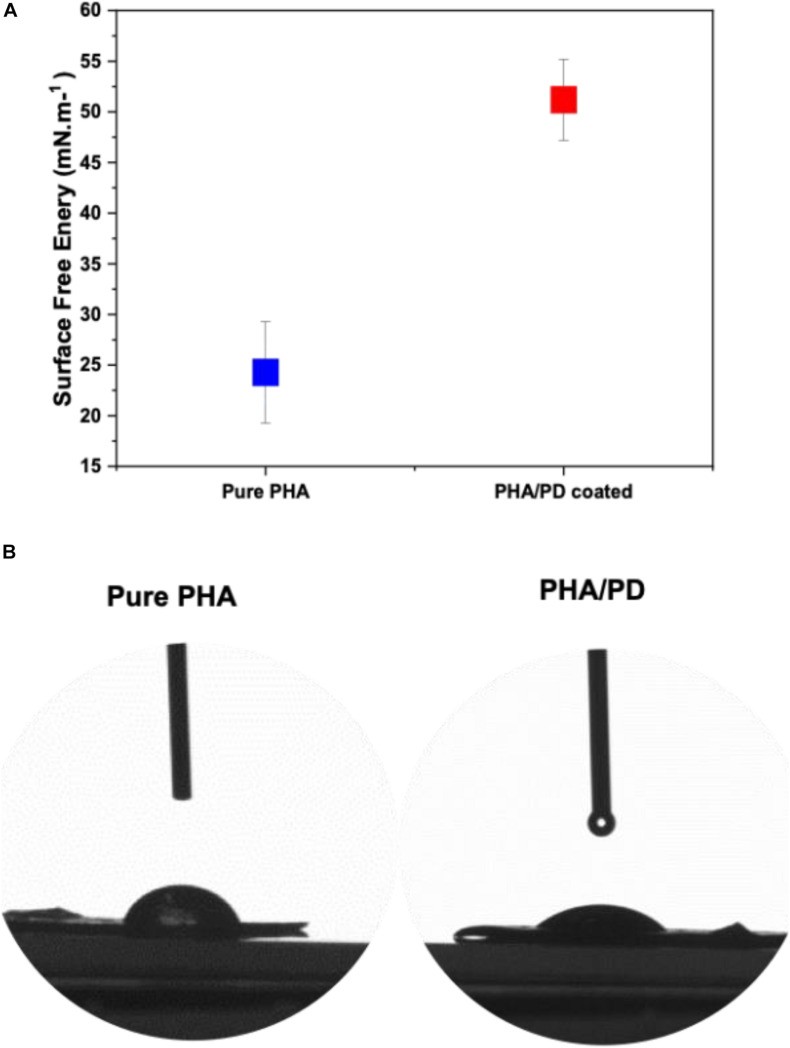
Contact angle measurements investigating the surface free energy of **(A)** pure PHA film compared to PHA/PD; there is a significant increase in the surface free energy value post PD immersion from 24.28 to 51.20 mN.m^–1^, *p* < 0.05. Furthermore, improved hydrophilicity of the polymer can be observed in **(B)** where an average water contact angle value of 49.69°± 4° was observed with the PD coating as opposed to 75°± 5° for the neat PHA.

The surface free energy of a material is essentially known as the variation of intermolecular bonds to form a surface or the excess energy which can indeed impact a materials wettability and its adhesion to the surrounding tissues (Tadmor et al., 2017). In this study, water, glycerol and diiodomethane were used to evaluate the surface free energy of the P(3HO-*co*-3HD-*co*-3HDD) films as a function of PD coating; there was a significant increase in the obtained value post PD immersion indicating a higher surface adhesion as a result of enhanced solid and liquid interaction (Figure 4A). Similarly, hydrophilicity of the polymer was found to have a direct correlation with PD surface functionalization, which can be clearly observed in the image shown in Figure 4.

**FIGURE 5:**
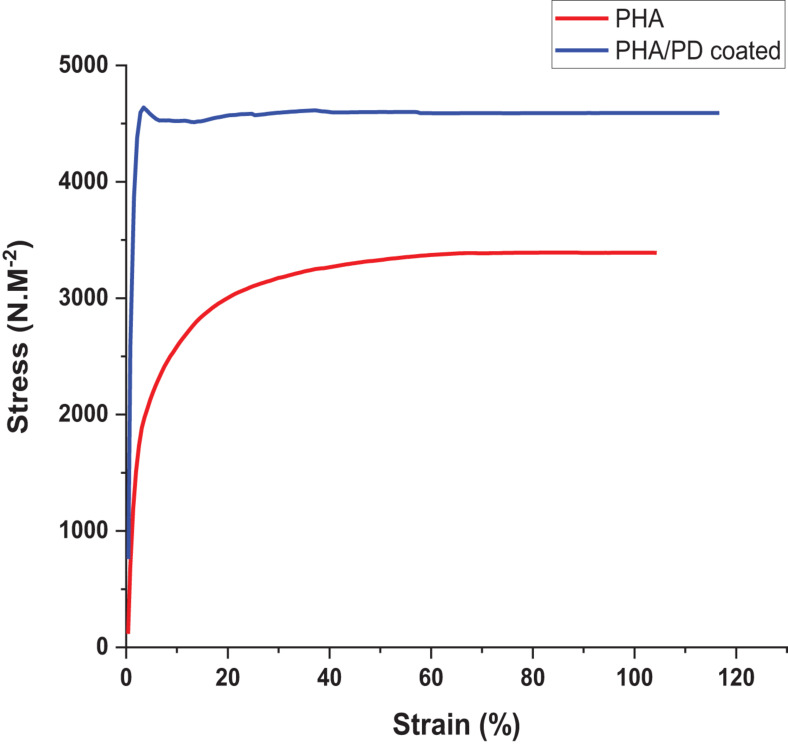
Reverse compression testing directly assessing adhesive properties of the samples. The force needed for detachment was measured and revealed increased adhesive properties for the polydopamine coated samples: PHA/PD exhibited a peak value of 4,500 N.M-2 which was significantly higher than pure PHA, *p* < 0.05.

In order to utilize a more direct method to assess the adhesiveness of the PHA films, as a result of PD surface functionalization, a reverse compression test was designed; this is a simple technique where an initial force was applied on the samples and quickly pulled back to the reverse direction. Therefore, the higher force required to detach from the plate directly correlated with an increased adhesiveness (“stickiness”) of the PHA patches (Rezaee et al., 2019). As shown in Figure 5, the force required to detach the PHA/PD required a peak value of 4,500 N.M^–2^ which was significantly higher the pure PHA (with a peak value of 3,000 N.M^–2^).

**FIGURE 6:**
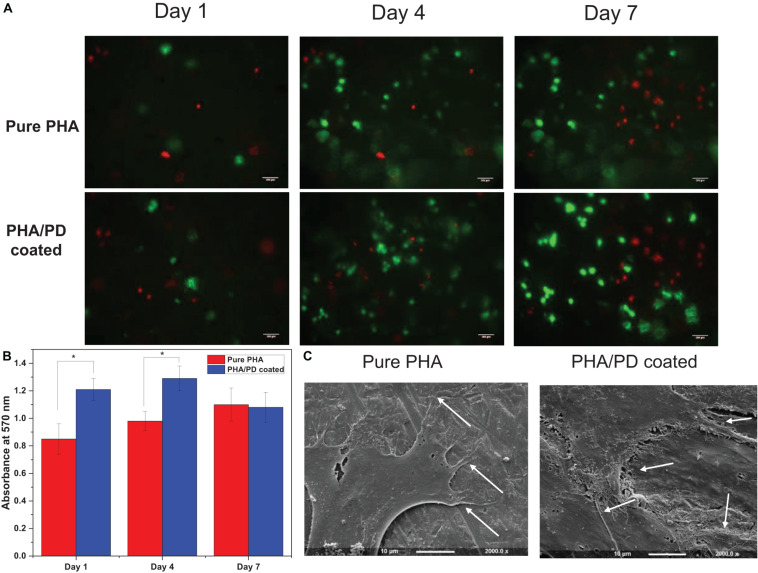
**(A)** Live/dead fluorescence images of fibroblasts on the polymer surface before and after polydopamine coating; this confirmed proliferation of the cells on pure PHA as well as PHA/PD across the 7-day incubation period. A clearer presence of cells can be noticed in the coated PHA samples by exhibiting strong green fluorescence after incubation. **(B)** Alamar blue activity of fibroblasts demonstrating higher absorbance values in coated samples compared to pure PHA. **(C)** Scanning Electron Microscopy images presenting cell interaction with the surface of the polymer films with a more flattened morphology post coating. The filopodia (identified by white arrows) are extending to increase contact with neighboring cells.

To further identify the effect of the PD coating, fibroblasts were seeded on the surface of the PD coated and uncoated PHA films. The Alamar blue assay allowed quantitative measurement of cell metabolism at 1-, 4-, and 7-days post incubation, where the coated films revealed a significantly higher absorbance value (Figure 6). This finding was consistent at day 1 and 4 of the incubation, as the coated samples showed absorbance values of 1.2–1.3 at 570 nm, respectively, compared to 0.85 and 0.95 on day 4 (*p* < 0.05). However, a slight drop in the absorbance values could be observed on day 7 for both films which could suggest reaching a state of confluency in cell proliferation (Abo-Aziza and ZakiA, 2017). Subsequently, attachment and viability of the cells were assessed via SEM microscopy and LIVE/DEAD imaging; SEM images (Figure 6C) demonstrate clear cell-material interactions following 1-day post surface functionalization. The difference between the coated and the non-coated surfaces involved formation of a clear monolayer of cells on the coated biofilm with an overall increase in cell processes as well as elongation (shown by white arrows in Figure 6). The cells presented a flattened morphology post coating where the filopodia was extending to increase contact with neighboring cells. This allows adhering to the underlying PHA film, which will in turn increase the cell–material interface area and ultimately enhance proliferation and migration (Zhao M. et al., 2020). The extracellular cues activated as a result of PD coating can account for this observation. Finally, LIVE/DEAD cell cytotoxicity assay allowed comparing viability of the cells on the surface of PHA films over the 7-day incubation period. Overall, a clearer presence of cells can be noticed in the coated PHA samples by exhibiting strong green fluorescence after incubation. Morphologically, the cells post PD coating appeared to be growing in clusters, however, the presence of dead cells, especially in day 7, could indicate a pH responsive response as a result of surface functionalization (Wang et al., 2019).

**FIGURE 7:**
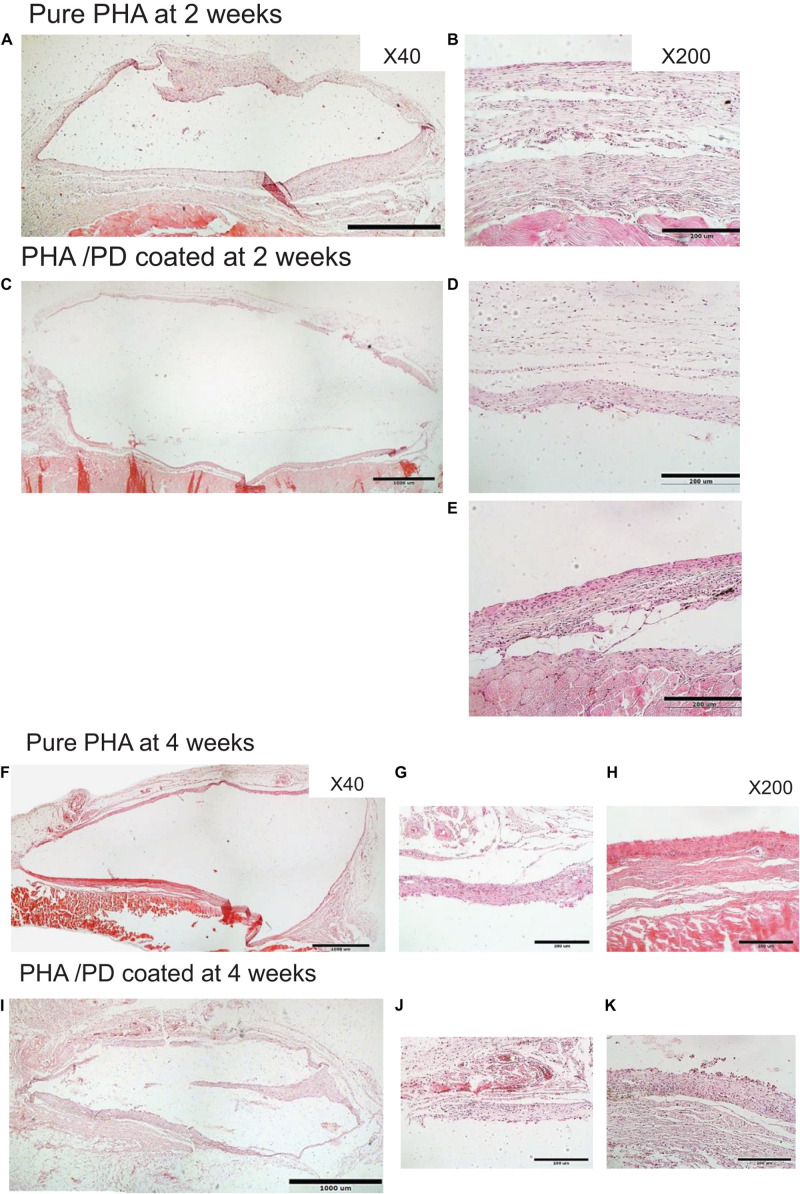
In order to investigate the *in vivo* biocompatibility of the coated PHA following simple PD immersion, the polymer films were implanted in a rat model subcutaneously. During the sacrifice, the state of the harvested tissues was stable with no visible sign of inflammatory response. H&E histological examination revealed that both implanted membranes were compatible at **(A–E)** 2 and **(F–K)** 4 weeks. However, the polymer interaction with the native tissue seems to have enhanced after PD coating, particularly at 4 weeks. Two magnifications were used to capture the images: ×40 shown in **A,C,F,I** and ×200 in **B,D,E,G,H,J,K**.

**FIGURE 8:**
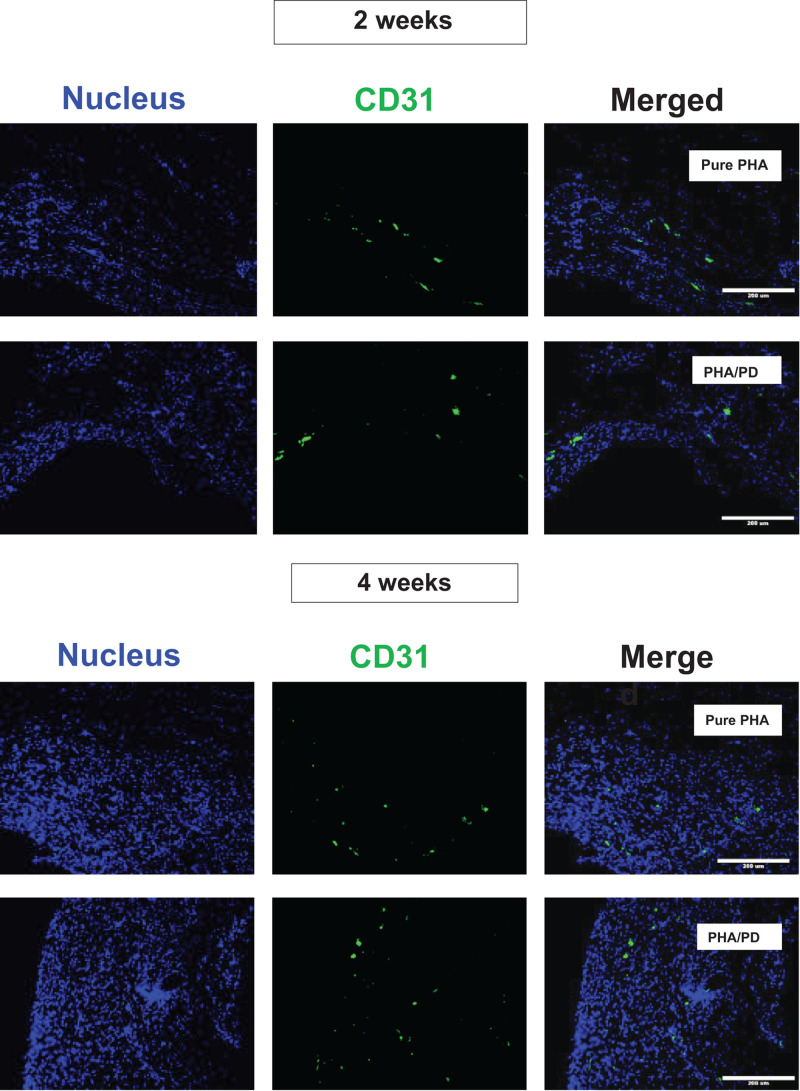
Assessment of CD31/DAPI markers 2- and 4-weeks post implantation of PHA and PHA/PD films to detect sign of neo-vascularization via endothelial cells: CD31 is shown in green, DAPI in blue and the merged image of the two stains can be observed on the right. Viable cells and sign of vascularization could be observed in both samples, with highest rates observed at week 4 post PD surface functionalization.

Finally, in order to evaluate the performance and biocompatibility of the PHA films, *in vivo* subcutaneous implantation was carried out in a rat model for the period of 2 and 4 weeks, respectively (Figure 7). During the sacrifice, the state of the tissues was stable with no visible signs of inflammation or fibrous invasions. This was followed by histological examination via H&E staining; both patches were found to be compatible at 2- and 4-weeks post implantation where a network of collagen fibers and cells were detected, shown in Figure 8. Comparing the two groups, the polydopamine loaded PHA patch exhibited less of an inflammatory response over time including a better integration with the native tissue. Moreover, signs of neovascularization were also observed around the PD coated PHA patches. To investigate this observation, CD31 immunostaining was carried out; accordingly, abundant presence of nuclei and expressed CD31 markers confirmed early signs of vascularization for both patches, which was more pronounced post surface functionalization, particularly at week 4.

## Discussion

Oral lesions, including oral lichen planus and aphthous ulcers are common in society and can impart a significant burden on patients’ quality of life. The treatment is often dependent on the use of corticosteroids, however, with poor drug delivery systems. Therefore, new ways of delivering these therapeutic agents are required to allow direct drug administration to the affected region. Controlled drug delivery to the oral mucosa has always been a significant challenge; this is due to moist mucosal surfaces coupled with salivary flow and abrasive forces within the oral cavity. In order to overcome the above-mentioned challenge, we used a muscle inspired polydopamine surface functionalization technique, developed by Lee et al. (2007). Surface functionalization via molecular design is a fundamental approach in incorporating novel functionalities into biomaterials for biomedical applications; the mussel inspired polydopamine contains similar functional groups to those of adhesive proteins including catechol and amine group and thus allowed to replicate the adhesive functionality via a very simple dopamine-based chemistry. The core of this technology is the extremely simple methodology, whereby spontaneous deposition of a conforming coating occurs during incubation and this first coat can be used “as is” or can be used as a primer for further modification. This simple coating method can be virtually applied to any substrate material, regardless of material chemistry, size and geometry (Tyo et al., 2019). The stability and robustness of the PD coating under different physiological conditions has made this technique particularly applicable to biomedical applications (Ding et al., 2016; Lee et al., 2007). Furthermore, this novel technique has been shown to enhance cell interactions with the material surface via introducing multiple functional groups (Luo et al., 2013), and consequently can be utilized as an effective surface functionalization method in biomaterials. In conjunction with this simple and novel surface coating technique, a group of naturally produced polymers, P(3HO-*co*-3HD-*co*-3HDD), were used in this study. PHAs are diverse bio polyesters synthesized by various bacteria via carbon as their energy source and have been produced in large quantity for several applications including medical implants. It has been demonstrated by many studies (Lim et al., 2017; Basnett et al., 2018; Elmowafy et al., 2019) that PHAs possess the required mechanical, biodegradable and tissue-compatible properties for biomedical applications. As a member of the polyhydroxyalkanoate (PHA) family, medium chain length PHAs have particularly attracted attention for a variety of medical applications due to their desirable properties such as high biocompatibility, low melting point and non-toxic degradation properties (Basnett et al., 2017; Boddupalli et al., 2010). More importantly the natural adhesive properties offered by the medium chain length PHAs provided a promising option as a mucosal patch for drug delivery (Bonartsev et al., 2019); Mucoadhesion is a complex phenomenon which involves wetting, adsorption, and interpenetration of polymer chains (Boddupalli et al., 2010). To achieve this, in the experimental work, followed by successful synthesis of P(3HO-*co*-3HD-*co*-3HDD) films, PD coating was carried out; initial assessment of cytocompatibility of these films revealed improved properties in terms of cell attachment and proliferation post surface functionalization. This was clear in cell viability values measured via metabolic activity within 7 days of incubation as well as enhanced cell integration in SEM microscopy with a flattened cell morphology. However, it must be noted that LIVE/DEAD imaging revealed a cluster of dead cells post PD coating. A recent study has indicated the cytocompatibility of PD coatings are cell type dependent (Ding et al., 2016); while Endothelial cells and chondrocytes exhibit highly enhanced adhesion and viability, limited megakaryocytic adhesion has been reported on PD coatings due to a pH sensitive response. Therefore, to further assess the biocompatibility of PD surface functionalization, *in vivo* subcutaneous implantation of the films was carried out at 2 and 4 weeks, respectively. Initial observation post retrieving the implants showed no visible sign of inflammation and fibrous invasions. Histological staining with H&E revealed the presence of visible nuclei in all samples, however, slightly in favor of PD coated films. CD31 was subsequently used to evaluate neo-vascularization, which is found on the surface of endothelial cells for monitoring of blood vessels. This is a critical factor in measuring optimum integration of foreign materials with native tissues as a result of enhanced bioactivity (Lee et al., 2015). Both coated and uncoated PHA films were observed to demonstrate a biocompatible behavior in post histological evaluation, though a more aligned spread of the marker was detected in the PHA/PD samples. Finally, for localized and controlled delivery, it is necessary to prolong and advance the contact between the drug and the mucosal lesion (Colley et al., 2018; Wang et al., 2018); this involves improving surface free energy as well as wettability. In this study, a PD coating was shown to be a powerful route in converting a bioinert substrate into a reasonably bioactive one; whereby the hydrophilicity of the polymer surface was improved, and a higher surface energy value was exhibited accordingly. This directly correlates with enhanced adhesions between the solid surface and the liquid drops and may be highly significant in drug administration. Furthermore, an adhesion test was established as a direct method via reverse compression measurements and revealed improved adhesiveness in PHA films as a result of PD coating.

## Conclusion

To conclude, it must be reiterated that the number of available polymers for successful fabrication of a mucoadhesive patch is very limited. To date most work has focused on the use of hydrogels, which are associated with both poor mechanical properties in terms of robustness and unpredictable degradation rates, resulting in material disintegration *in vivo*. Microbially synthesized PHAs have been shown to degrade slowly via surface erosion into non-toxic biodegradable metabolites (Koller, 2018). Therefore, the novel PD surface coating technique coupled with the use of PHAs hold promising insight in establishing a long-lasting potential oral patch with localized drug delivery. The PHA/PD coated films revealed promising results in this study with improved surface free energy, hydrophilicity and adhesiveness as well as enhanced *in vitro* cell proliferation and *in vivo* neo-vascularization. However, to further verify the suitability of this system for effective clinical translation, future work needs to focus on scaling up the production of PHA films as well as loading an appropriate drug for the ultimate aim of sustained local release.

## Data Availability Statement

The raw data supporting the conclusions of this article will be made available by the authors, without undue reservation.

## Ethics Statement

The animal study was reviewed and approved by the Dankook University Ethics committee DKU-18-032.

## Author Contributions

NO, DG, NM, and EM carried out the laboratory work. All authors designed the experiments and edited the manuscript and figures.

## Conflict of Interest

The authors declare that the research was conducted in the absence of any commercial or financial relationships that could be construed as a potential conflict of interest.
